# The effectiveness and safety of proton beam radiation therapy in children with malignant central nervous system (CNS) tumours: protocol for a systematic review

**DOI:** 10.1186/s13643-016-0285-6

**Published:** 2016-07-27

**Authors:** Caroline Main, Madhumita Dandapani, Mark Pritchard, Rachel Dodds, Simon P. Stevens, Nicky Thorp, Roger E. Taylor, Keith Wheatley, Barry Pizer, Matthew Morrall, Robert Phillips, Martin English, Pamela R. Kearns, Sophie Wilne, Jayne S. Wilson

**Affiliations:** 1 Cancer Research UK Clinical Trials Unit (CRCTU), Institute of Cancer and Genomic Science, University of Birmingham, Birmingham, UK; 2 Birmingham Children’s Hospital NHS Foundation Trust, Birmingham, UK; 3 Royal Stoke University Hospital, Stroke-on-Trent, UK; 4 Leeds General Infirmary, Leeds, UK; 5 The Clatterbridge Cancer Centre, Liverpool, UK; 6 Swansea University, Swansea, UK; 7 Alder Hey Children’s NHS Foundation Trust, Liverpool, UK; 8 Centre for Reviews and Dissemination (CRD), University of York, York, UK; 9 Queen’s Medical Centre, Nottingham University Hospitals’ NHS Trust, Nottingham, UK

**Keywords:** Children, Central nervous system tumours, Proton beam RT, Conventional photon RT, Systematic reviews

## Abstract

**Background:**

The aim of this study is to use a systematic review framework to identify and synthesise the evidence on the use of proton beam therapy (PBT) for the treatment of children with CNS tumours and where possible compare this to the use of photon radiotherapy (RT).

**Methods:**

Standard systematic review methods aimed at minimising bias will be employed for study identification, selection and data extraction. Twelve electronic databases have been searched, and further citation, hand searching and reference checking will be employed. Studies assessing the effects of PBT used either alone or as part of a multimodality treatment regimen in children with CNS tumours will be included. Relevant economic evaluations will also be identified. The outcomes are survival (overall, progression-free, event-free, disease-free), local and regional control rates, short- and long-term adverse events, functional status measures and quality of survival. Two reviewers will independently screen and select studies for inclusion in the review. All interventional study designs will be eligible for inclusion in the review. However, initial scoping searches indicate the evidence base is likely to be limited to case series studies, with no studies of a higher quality being identified. Quality assessment will be undertaken using pre-specified criteria and tailored to study design if applicable. Studies will be combined using a narrative synthesis, with differences in results between studies highlighted and discussed in relation to the patient population, intervention and study quality. Where appropriate, if no studies of a comparative design are identified, outcomes will be compared against a range of estimates from the literature for similar populations and treatment regimens from the best available evidence from studies that include the use of advanced conventional photon therapy.

**Discussion:**

The evidence base for the use of PBT in children with CNS tumours is likely to be relatively sparse, highly heterogeneous and potentially of a low quality with small sample sizes. Furthermore, selection and publication biases may limit the internal and external validity of studies. However, any tentative results from the review on potential treatment effects can be used to plan better quality research studies that are of a design appropriate for outcome comparison with conventional therapy.

**Systematic review registration:**

PROSPERO CRD42015029583

**Electronic supplementary material:**

The online version of this article (doi:10.1186/s13643-016-0285-6) contains supplementary material, which is available to authorized users.

## Background

Tumours of the central nervous system (CNS) represent a group of neoplasms that account for approximately 25 % of all childhood cancers. These tumours are both anatomically and histologically diverse. They are the leading cause of cancer-related death in childhood, and whilst it has become clear that the use of multimodal treatment regimens including surgery, radiotherapy (RT) and chemotherapy can increase survival, 60 % of survivors are moderately or severely disabled due to the disease and treatment-related effects [1]. With more individuals having the prospect of long-term survival, the focus of treatment has therefore started to shift from one of ‘cure at all costs’ to ‘the cost of cure’. Novel treatment strategies are therefore being introduced in an attempt to maintain or increase survival rates whilst maximising the quality of the resultant long-term survival. Proton beam therapy (PBT) is one such treatment approach.

Conventional photon RT used primarily as a local therapy aims to provide improved local tumour control and cure whilst minimising radiation doses to adjacent normal tissues. However, the appropriate targeting and delivery of radiation dose is complex and is particularly difficult for tumours adjacent to radiation-sensitive critical body structures (the so-called organs at risk) such as the pituitary, optic chiasm, hippocampus, lungs, bowel, ovaries, heart and thyroid gland. For this reason, in clinical practice, tumour lethal doses are not always achieved due to the need to balance the desired damage to the tumour with the undesirable radiation-induced injury to adjacent healthy tissues [2]. This is generally achieved by targeting the beam to the tumour area through paths that spare nearby critical and radiosensitive anatomical structures, selecting multiple fields that cross in the tumour area through different paths, and splitting the total dose into multiple smaller dose ‘fractions’ that are delivered over several days to weeks, thereby allowing damage to normal tissues to recover between treatments.

PBT is a form of RT that delivers radiation within a defined radiation track length, with virtually no dose beyond the intended target [3]. In contrast to conventional RT, where potentially larger volumes of healthy tissue are irradiated, PBT is associated with smaller normal tissue-irradiated volumes [4–8], decreasing the dose to healthy tissues by a factor of 1.5 to 3.0 mainly due to the generally lower entrance dose and the complete elimination of exit dose compared to photon beams [9].

It therefore allows the benefit of a more localised delivery of RT that can be exploited either by targeting higher RT doses to the tumour without increased RT-induced normal tissue toxicity or by reducing adverse effects at equally effective doses. At the present time, most PBT is delivered using three-dimensional conformal techniques (3D-CPT) via the passive beam scattering method. However, proton pencil beam scanning (PBS) techniques are gradually being introduced at several institutions [10], with these allowing for delivery of intensity-modulated proton therapy (IMPT) and the potential to further improve the target conformity and sparing of adjacent healthy organs.

The use of PBT in the treatment of children with CNS tumours is particularly appealing given the fact that some tumours may not be surgically operable, the radiosensitivity of the brain and spinal cord, and the need to minimise the deleterious effects of RT on the developing CNS. Secondary malignancies and late effects of treatment are of particular concern in long-term survivors of childhood CNS tumours following conventional RT, and results of dose planning studies have raised the question as to whether the improved dose distribution in proton therapy may reduce the risk of secondary malignancies [7, 8].

To date, a number of case series studies have assessed the effectiveness, safety or long-term treatment sequelae of the use of proton RT in children with different malignant CNS tumours. These have included children with low-grade gliomas (LGG) [11, 12], ependymoma [13], germ cell tumours [14], pineoblastoma [15] and atypical teratoid/rhabdoid tumours (AT/RT) [16]. Whilst three systematic reviews have previously been conducted on the use of PBT, these have included both children and adults with all types of cancer diagnoses and were published in 2007 [17, 18] and 2009 [19]. They therefore do not provide a comprehensive up-to-date assessment of the use of PBT in the treatment of children with CNS tumours. Additionally, two recent non-systematic literature reviews highlighting further studies on the effects of PBT in the treatment of children with CNS tumours have now been undertaken [20, 21], and therefore, there is a need to provide a systematic up-to-date review of the evidence relating to the use of this technology in children with CNS tumours.

However, all of these reviews highlight the lack of the evidence for comparing the effects of PBT with conventional or advanced photon RT [17–21], with nearly all the evidence based on small, single-group, retrospective case series studies. It is acknowledged within the present proposed review that this is not an appropriate design for comparing the relative effects of PBT and conventional RT, and therefore, the aim of the review is to use a transparent, replicable systematic review framework based on the ‘best-evidence’ approach [22, 23] in order to identify and synthesise the available evidence on the impact of treatment with PBT in children with CNS tumours.

## Methods

Standard systematic review methodology aimed at minimising bias will be employed, and reporting will follow the Preferred Reporting Items for Systematic Reviews and Meta-Analyses (PRISMA) guidelines [24]. The protocol for this review is registered with PROSPERO (CRD42015029583), available from http://www.crd.york.ac.uk/PROSPERO/display_record.asp?ID=CRD42015029583. The Preferred Reporting Items for Systematic Review and Meta-Analysis Protocols (PRISMA-P) checklist for the review is also included as Additional file 1.

### Data sources and searches

This review forms part of a wider work programme of systematic reviews that aim to assess the effects of different interventions for the treatment of CNS tumours in children, adolescents and young adults. Searches have therefore been conducted for studies examining the effects of surgery, radiotherapy, chemotherapy, immunotherapy, hormone therapy, biological therapies and imaging used alone or as part of a multimodality treatment regimen for all types of paediatric brain tumours. No study design filters have been applied to the searches. Specific details of the searches conducted are detailed below.

#### Bibliographic databases

A comprehensive, broad search strategy was developed using a combination of Medical Subject Headings (MeSH) and free-text terms. The searches were limited by date from 1985 to November week 1, 2014, and updated in November week 1, 2015. No language or publication status restrictions were applied, and ongoing studies were included.

The searches for published studies were undertaken using the following databases: MEDLINE (OvidSP); MEDLINE In-Process Citations and Daily Update (OvidSP); EMBASE (OvidSP); Cochrane Database of Systematic Reviews (CDSR) (Wiley); Cochrane Central Register of Controlled Trials (CENTRAL) (Wiley); CINAHL Plus (EBSCO); PsycINFO (OvidSP); NHS Economic Evaluation Database (NHS EED) (CRD website), DARE (CRD website); and HTA (CRD website). The search strategy used for the MEDLINE search is reported in the Appendix.

#### Grey literature

Completed and ongoing studies were identified by searches of NIH Clinical Trials (http://www.clinicaltrials.gov/); Current Controlled Trials (http://www.controlled-trials.com/); and WHO International Clinical Trials Registry Platform (ICTRP) (http://www.who.int/ictrp/en/).

#### Other sources

Experts in the field, from both the Project Advisory and Patient and Public Involvement (PPI) Groups, were contacted with a list of identified studies to find out whether they had knowledge of any further studies that had not been retrieved by the electronic searches. Reference lists of all studies included in the present review will be checked, citation searching will be undertaken and the following books of conference abstracts will be hand-searched:

- *Annual Meeting of the American Society for Radiation Oncology* (ASTRO) (56th and 57th meeting abstracts)
- *International Society of Paediatric Oncology* (SIOP) (46th and 47th meeting abstracts)
- *International Symposium on Pediatric Neuro-Oncology* (ISPNO) (15th and 16th meeting abstracts)
- *American Society of Clinical Oncology* (ASCO) (2014 and 2015)

All identified references have been downloaded into Endnote X7 software for initial assessment and handling. Where flexibility is needed throughout the work programme for reference management and handling, Endnote software will be linked to bespoke Access databases in order to facilitate sorting and manipulation of data items within indexed fields and abstracts. As a preliminary first stage to the broader set of reviews, and as an aid to the research question prioritisation process, inclusion screening on the basis of population and the broader set of applicable interventions has already been undertaken, with all included studies being ‘mapped’ by study design.

### Study selection

All studies have been loosely ‘tagged’ according to the study design and type of intervention using the seven intervention categories outlined above. All studies ‘tagged’ as proton therapy will be used to form the potential pool from which studies will be screened against the specific inclusion criteria. Study selection will be undertaken by two reviewers working independently initially using the titles/abstracts from the pool of potential studies. Studies marked for inclusion by either reviewer will then undergo full independent text assessment. Any discrepancies will be resolved by recourse to the abstracts or full texts or through consensus with a third reviewer. A PRISMA flow chart illustrating the study selection process will be documented [24].

### Inclusion/exclusion criteria

#### Population

The study population covers infants, children and young adults (up to approximately age 25 years) with diagnoses of any type of malignant CNS tumour. These include but are not limited to high- and low-grade gliomas (HGG and LGG), diffuse intrinsic pontine glioma (DIPG), medulloblastoma, ependymoma, germ cell tumours, atypical teratoid/rhabdoid tumour (AT/RT), primitive neuroectodermal tumours (PNET) and pineoblastoma. Studies that include both children and adults within the relevant populations or that include children with different types of CNS tumours will be included provided that participant baseline demographic data and outcomes are reported separately for children by CNS tumour type.

#### Interventions

Interventions include PBT used either alone or as part of a multimodality treatment regimen that may include conventional photon external beam radiation.

#### Comparator (for controlled studies)

The comparator used in this study is conventional photon external beam radiation including 3D conformal techniques or intensity-modulated radiotherapy (IMRT) including arc therapy, stereotactic radiosurgery or brachytherapy used alone or as part of a multimodality treatment programme. Data on the range of outcome estimates for use of the most advanced types of photon radiation in each tumour type included in the review will be sought (if necessary) in order to allow an informal comparison with the outcome estimates from studies included in the review if no controlled studies of PBT are identified.

#### Outcomes

The outcomes are overall survival (OS), surrogate survival outcomes (progression-free (PFS), disease-free (DFS), event-free (EFS)), local control, regional control, short- and long-term adverse events, functional status measures (including neurocognitive and educational outcomes) and quality of survival. Studies that only report changes on magnetic resonance images (MRI) or evaluate treatment planning or dosimetry without providing any data on clinical outcomes or adverse events will be excluded.

#### Study designs

The study designs include randomised controlled trials (RCTs), non-randomised trials, phase II single-arm trials, prospective and retrospective case series and ongoing trials. Relevant economic evaluations will also be identified. Cross-sectional studies and multiple and single case reports will be excluded.

### Data extraction

Data will be recorded on a standard data extraction form developed in either Access or Excel. The data will be extracted by one reviewer and checked by a second for accuracy. Any discrepancies will be resolved by recourse to the paper. Data from studies with multiple publications will be extracted and reported as a single study.

Data will be extracted on the following: general information (study name, study group (if applicable), publication date(s), principal investigator/authors); patient eligibility and study participants (e.g. tumour type and location, grade, age, gender, prior treatment history); RT image planning (e.g. computerised tomography (CT) or MRI, imaging plane and weighting, image contrast enhancement); definition of clinical tumour volume and margins; intervention and comparator (where applicable): RT (type, dosimetry (Gy), fractions, field arrangement and number of fields, concomitant therapy), number of administrations and time frame, any supportive care, treatment intent (radical or palliative), study design (e.g. controlled trial, single-arm phase II trial or case series), length of follow-up and timing of outcome assessments; outcome measures (protocol specified—if available—and reported); side effects/toxicity, long-term adverse events and neuropsychological outcomes; analysis methods (ITT or per protocol); and the author’s conclusions. If any controlled studies are identified, outcomes will be recorded separately for controlled and observational studies.

### Assessment of risk of bias in studies

If there are any RCTs identified, study quality will be assessed using the Cochrane Risk of Bias tool [25]. It is, however, anticipated that the research evidence will be non-comparative in nature; therefore, to assess the risk of bias, the checklist adapted by Wilson and colleagues [26] based upon the six-point checklist developed by CRD, York, for the assessment of observational studies will be used (http://www.york.ac.uk/crd/SysRev/!SSL!/WebHelp/SysRev3.htm) [27]. This covers the domains of selection, detection and attrition bias. Additional criteria will also be used to assess the adequacy of the sample size and methods of analyses, whether all assessed outcomes are reported, and the likely external validity of the study. All assessments will be at the overall study level, not at the level of the individual outcomes. Quality assessment will be undertaken by one reviewer and independently checked for accuracy by a second. Any disagreements will be resolved by recourse to the study paper(s), and a third reviewer will be consulted where necessary.

### Data synthesis and analysis

A narrative synthesis of study results will be presented (including text, figures and tables), to provide adequate interpretation of study findings. The initial narrative syntheses from the review team will be developed, discussed, refined and tested iteratively with clinical members of the team until a final consensus or conclusion is reached. Where appropriate, the summary figure for outcomes will be expressed as a weighted mean (weighted by the initial sample size). Studies will be grouped by tumour type, by treatment line (induction, consolidation, salvage) and then by the intervention (where this differs significantly between studies). Differences between the studies will be highlighted and discussed in relation to the patient population, intervention and study quality. Where appropriate (and no comparative studies are identified), outcomes for studies will be compared against a range of estimates from the literature for similar populations and treatment regimens that include the use of the most relevant and up-to-date advanced conventional photon therapy as part of the multimodal treatment regimen to provide a context for the interpretation of results.

Where more than one RCT has addressed the same question and they are considered to be clinically similar (based on patient population and study treatments), results will be combined in a standard pairwise meta-analysis using assumption-free methods. All analyses will be carried out on an intention-to-treat (ITT) basis where possible, using the HR or RR as appropriate. Heterogeneity of treatment effect, if present, will be investigated using the chi-squared test for heterogeneity and the *I*^2^ statistic [28]. Further sub-group analyses, to explore differences between the trials in terms of patient baseline characteristics such as tumour grade and prior treatments (dose, number of cycles), will be undertaken as necessary to investigate whether the treatment effect differs between patient sub-groups.

An overview of the results and the range of costs of any up-to-date, robust economic evaluations and the limitations of the existing models will be presented in the ‘Discussion’ section.

## Discussion

Although this methodology has been designed to be comprehensive and to minimise bias, any results are likely to be highly tentative and context specific. The evidence base is likely to be sparse, highly heterogeneous and of a low quality with small sample sizes. Furthermore, the included studies are likely to be subject to selection bias, as PBT is only conducted at a limited number of highly specialised centres. Furthermore, access to PBT especially within the USA is linked to insurance status, and the treatment of patients with different clinical indications may potentially be associated with whether PBT facilities are run on a for-profit or non-profit basis. This means that the external validity of the studies is likely to be low, with patients entered into the studies differing from those that might generally be seen in practice. It is therefore posited that the ‘noise’ to ‘signal’ ratio within the data will be high, and it will be difficult to delineate any potential treatment effects. Moreover, there is strong potential for significant publication bias within the review due to the poor indexing of case series studies, and the lack of any requirement, or indeed web-based portal on which to register non-comparative studies. There will therefore be a strong reliance on identifying potentially relevant literature through ‘snowballing’ techniques, reference checking, citation searching and contact with experts in the field to identify studies that have only recently been completed. However, given the impact of the introduction of PBT within the UK by 2018 with significant financial resources being invested in facilities, and the impact that this might have for the treatment of children with CNS tumours, it is important to undertake this review despite the potential methodological limitations and caveats outlined above. Moreover, the assembly of evidence on the potential impact of different treatment settings and across different sub-populations can be used to make informed decisions regarding the likely impact of the implementation of the technology within new settings and be used to plan better quality research studies that are appropriate for outcome comparison with advanced conventional therapy.

To ensure that our findings have clinical impact on patients, their parents and the physicians who care for them, results will be disseminated broadly by presenting at scientific conferences, publishing in peer-reviewed journals and through our established PPI Partners who are associated with high-profile brain tumour charities.

## Abbreviations

AT/RT, atypical teratoid/rhabdoid tumour—an embryonal tumour that can occur anywhere in the central nervous system; CNS, central nervous system—the part of the nervous system consisting of the brain and the spinal cord; CT, computerised tomography—radiography in which a three-dimensional image of a body structure is constructed by computer from a series of plane cross-sectional images made along an axis; Gy, the gray—a derived unit of ionising radiation dose defined as the absorption of 1 J of radiation energy per 1 kg of matter; IMRT, intensity-modulated radiotherapy—an advanced high-precision type of radiotherapy that uses computer-controlled linear accelerators to deliver precise radiation doses that conform to the shape of a tumour or specific area within a tumour; LGG, low-grade gliomas—tumours that exhibit glial differentiation and lack high-grade findings such as microvascular proliferation and necrosis; MRI, magnetic resonance imaging—a technique that uses a magnetic field and radio waves to create detailed images of the organs and tissues within the body; PBS, proton pencil beam scanning—a type of proton beam therapy in which a lot of small pencil beams are directed into the target in order to cover the 3D volume; PBT, proton beam therapy—a type of radiotherapy that uses proton beams as opposed to standard photon radiotherapy that uses X-ray beams composed of primary photons and secondary electrons

## Appendix

### Clinical Effectiveness Search Strategy

Medline (OvidSP): 1985- October Week 4 2014

1. Glioma/or Brain Neoplasms/or Meningioma/or Glioblastoma/or Astrocytoma/
2. ((brain or brainstem or intracranial or posterior fossa) adj3 (cancer* or carcinom* or tumor* or tumour* or neoplasm*)).mp.
3. (Astrocytoma* or Brain Stem Glioma* or Medulloblastoma*or Primitive Neuroectodermal Tumo?r* or ganglioneuroblastoma* or CNS neuroblastoma* or Ependymoblastoma or Medulloepithelioma or Pineal Parenchymal Tumour* or (Atypical Teratoid adj1 tumo?r*) or Oligoastrocytoma or ((Pilocytic or Gemistocytic) adj1 astrocytoma*) or ependymoma or primitive neuroectal tumo?r*).mp.
4. (((Diffuse fibrillary or Gemistocytic or Pilocytic Pilomyxoid Protoplasmic Subependymal giant cell) adj1 astrocytoma*) or Oligoastrocytoma or Oligodendroglioma or Oligoastrocytoma or Pleomorphic xanthoastrocytoma or ((astrocytoma or oligoastrocytoma or oligodendroglioma) adj1 astrocytoma*) or Glioblastoma or Gliomatosis cerebri or Gliosarcoma or ((diffuse intrinsic pontine glioma or low grade brain stem) adj1 glioma) or ((classic or desmoplastic or nodular or large cell or nodularity) adj1 medulloblastoma*) or Primitive Neuroectodermal Tumo?r* or ((ganglioneuroblastoma or neuroblastoma) adj 1central nervous system*) or Ependymoblastoma or Pineoblastoma or pineal parenchymal tumo?r* or (central nervous system adj1 atypical teratoid) or (central nervous system adj 1 rhabdoid tumo?r*) or Germinomas or ((immature or mature or malignant transformation) adj2 teratomas)).mp.
5. 1 or 2 or 3 or 4
6. exp Surgical Procedures, Operative/
7. surg*.mp.
8. debulk*.mp.
9. cytoreduc*.mp.
10. 6 or 7 or 8 or 9
11. (chemotherap* or antineoplastic agents or cytotoxic or alkylating agents or nitrosoureas or antimetabolite* or antitumor?r or ((antibod* or monoclonal) adj 3 Human*) or plant alkyloid* or (hormone* adj 1 agent*) or anthracycline* * or systemic therap*).mp.
12. (Everolimus or Afinitor or Cetuximab or Erbitux or Bevacizumab or Avastin or Cediranib or Recentin or lomustine or CCNU or CeeNU or carmustine or BiCNU or Carustine or Ethylnitrosourea or Streptozocin or Sorafenib or Nexavar or tipifarnib or Zarnestra or Erlotinib or Tarceva or Sorafenib or Nexavar or temsirolimus or Torisel or Sunitinib or Sutent or irinotecan or Camptosar or Campto or Vandetanib or Caprelsa or Cabozantinib or Cometriq or XL184 or Axitinib or AG013736 or Inlyta).mp.
13. 11 or 12
14. exp Immunotherapy/ae, cl, ct, mt, mo, nu, px, st
15. exp Genetic Therapy/ae, cl, ct, mt, mo, nu, ut
16. exp Imaging, Three-Dimensional/or exp Whole Body Imaging/or exp Magnetic Resonance Imaging/
17. exp Tomography, Emission-Computed/or exp Four-Dimensional Computed Tomography/or exp Tomography/or exp Tomography, Emission-Computed, Single-Photon/or exp Positron-Emission Tomography/
18. 16 or 17
19. (radiation therapy or radiotherap* or intensity modul at* radiotherapy*or radiosurgery or radiation oncology or reduced boost volume radiotherap* or hyper fractionat* stereotactic radiotherap*or adjuvant radiotherap* or body radiotherap* stereotactic*or computer assisted radiotherap*or computer assisted radiotherap*planning or conformal radiotherap* or dosage* radiotherap* or dose fractionation* radiotherap* or high energy radiotherap*or implant radiotherap*or intensity or modulated radiotherap*or interstitial radiotherap*orimage guided radiotherap*or stereotactic*guid* radiotherap* or local therap* or proton therap* or proton adj2 therap* or proton beam therap* or proton adj2 radiation or proton radiation therap* or proton adj2 radiotherap* or proton adj2 irradia* or PBT).mp.
20. 10 or 13 or 14 or 15 or 18 or 19
21. 5 and 20
22. (Response or overall survival or progression* free survival or event* free survival or time to recurrence or time to progression or disease* free interval* or endocrinopath* or ((growth or thyroid) adj 1 hormone adj 3 deficienc*) or ((glucocorticoid or gonadotropin) adj 3 deficienc*) or endocrine dysfuct* or (cardiac function* adj 3 impair*) or ataxia or spastic paresis or visual dysfunction or epilepsy or hemiparesis or neurolog* deficit*).mp.
23. 21 and 22
24. limit 23 to (yr = “1985 -Current” and (“newborn infant (birth to 1 month)” or “infant (1 to 23 months)” or “preschool child (2 to 5 years)” or “child (6 to 12 years)” or “adolescent (13 to 18 years)” or “young adult (19 to 24 years)”) and humans)

## Additional file

Additional file 1: Preferred Reporting Items for Systematic Reviews and Meta-Analysis Protocols (PRISMA-P) 2015 checklist for the effectiveness and safety of proton beam radiation therapy in children with malignant central nervous system (CNS) tumours.

## References

[1] Boman K, Hoven E, Sinclair M, Lannering B, Gustafsson G (2009). Health and persistent functional late effects in adult survivors of childhood CNS tumours: a population-based cohort study. Eur J Cancer.

[2] Levin WP, Kooy H, Loeffler JS, DeLaney TF (2005). Proton beam therapy. Br J Cancer.

[3] Lundkvist J, Ekman M, Ericsson SR, Jonsson B, Glimelius B (2005). Proton therapy of cancer: potential clinical advantages and cost-effectiveness. Acta Oncol.

[4] Lee CT, Bilton SD, Famiglietti RM, Riley BA, Mahajan A, Change EL (2005). Treatment planning with protons for pediatric retinoblastoma, medulloblastoma, and pelvic sarcoma: how do protons compare with other conformal techniques?. Int J Radiat Oncol Biol Phys.

[5] Weber DC, Rutz HP, Lomax AJ, Schneider U, Lombriser N, Zenhausem R (2004). First spinal axis segment irradiation with spot-scanning proton beam delivered in the treatment of a lumbar primitive neuroectodermal tumour. Case report and review of the literature. Clin Oncol (R Coll Radiol).

[6] Glimelius B, Isacsson U, Blomquist E, Grusell E, Jung B, Montelius A (1999). Potential gains using high-energy protons for therapy of malignant tumour. Acta Oncol.

[7] Hall EJ (2006). Intensity-modulated radiation therapy, protons, and the risk of second cancers. Int J Radiat Oncol Biol Phys.

[8] Mu X, Bjork-Eriksson T, Nill S, Oelfke U, Johansson KA, Gagliardi G (2005). Does electron and proton therapy reduce the risk of radiation induced cancer after spinal irradiation for childhood medulloblastoma? A comparative treatment planning study. Acta Oncol.

[9] Paganetti H, van Luijk P (2013). Biological considerations when comparing proton therapy with photon therapy. Semin Radiat Oncol.

[10] Clasie B, Depauw N, Fransen M, Goma C, Panahandeh HR, Seco J, Flanz JB, Kooy HM (2012). Golden beam data for proton pencil-beam scanning. Phys Med Biol.

[11] Greenberger BA, Pulsifer MB, Ebb DH, MacDonald SM, Jones RM, Butler WE, Huang M, S, Marcus KJ, Oberg JA, Tarbell NJ, Yock TI. Clinical outcomes and late endocrine, neurocognitive, and visual profiles of proton radiation for pediatric low-grade gliomas. Int J Radiat Oncol Biol Phys. 2014;89 (5):1060-8. 10.1016/j.ijrobp.2014.04.053 25035209

[12] Indelicato DJ, Rotondo RL, Flampouri S, Sandler E, Aldana P, Mendenhall NP, Marcus RB (2012). Proton therapy for low grade glioma: early clinical outcomes. Pediatric Blood Cancer.

[13] Mac Donald SM, Sethi R, Lavally B, Yeap BY, Marcus KJ, Caruso P, Pulsifer M, Huang M, Ebb D, Tarbell NJ, Yock TI. Proton radiotherapy for pediatric central nervous system ependymoma: clinical outcomes for 70 patients. Neuro-Oncol. 2013, 15 (11), 1552-559 10.1093/neuonc/not121 PMC3813421 24101739

[14] MacDonald SM, Trofimov A, Safai S, Adams J, Fullerton B, Ebb D, Tarbell NJ, Yock TI (2011). Proton radiotherapy for pediatric central nervous system germ cell tumors: early clinical outcomes. Int J Radiat Oncol Biol Phys.

[15] Farnia B, Allen PK, Brown PD, Khatua S, Levine NB, Li J, Penas-Prado M, Mahajan A, Ghia AJ (2014). Clinical outcomes and patterns of failure in pineoblastoma: a 30-year, ingle-institution retrospective review. World Neurosurg.

[16] McGovern SL, Okcu M, Munsell M, Grosshans D, McAleer MF, Chintagumpala M, Khatua S, Mahajan A (2014). Proton therapy for pediatric AT/RT of the CNS. Int J Radiat Oncol Biol Phys.

[17] Olsen D, Bruland O, Frykholm G, Norderhaug I. Proton therapy—a systematic review of clinical effectiveness. 2007, 83, 123-32. 10.1016/j.radonc.2007.03.001 17499374

[18] Brada M, Pijls-Johannesma M, De Ruysscher D (2007). Proton therapy in current practice: current clinical evidence. J Clin Oncol.

[19] Terasawa T, Dvorak T, Ip S, Raman G, Lau J, Trikalinos T (2009). Systematic review: charged-particle radiation therapy for cancer. Ann Intern Med.

[20] Rombi B, Vennarini S, Vinante L, Ravanelli D, Amichetti M (2014). Proton radiotherapy for pediatric tumors: review of first clinical results. Ital J Pediatr.

[21] Mahajan A (2014). Proton craniospinal radiation therapy: rationale and clinical evidence. Int J Particle Therapy.

[22] Treadwell JR, Singh S, Talati R, McPheeters ML, Reston JT (2012). A framework for best evidence approaches can improve the transparency of systematic reviews. J Clin Epidemiol.

[23] Norris S, Atkins D, BrueningWEA. Selecting observational studies for comparing medical interventions; Rockville, MD, 2010. 21433401

[24] Moher D, Liberati A, Tetzlaff J, Altman DG (2009). Preferred Reporting Items for Systematic Reviews and Meta-Analyses: the PRISMA statement. Ann Intern Med.

[25] Higgins JPT, Altman DG, Sterne JAC. Chapter 8: Assessing risk of bias in included studies. 2011. In: Cochrane handbook for systematic reviews of interventions version 510 (updated March 2011) [Internet]. The Cochrane Collaboration. Available from: http://handbook.cochrane.org/. Accessed 21 Nov 2015.

[26] Wilson JS, Gains JE, Moroz V, Wheatley K, Gaze MN (2014). A systematic review of 131I-meta iodobenzylguanidine molecular radiotherapy for neuroblastoma. Eur J Cancer.

[27] Centre for Reviews and Dissemination (CRD). Systematic reviews: CRD’s guidance for undertaking reviews in health care 2008. Available from: https://www.york.ac.uk/crd/guidance/. Accessed 21 Nov 2015.

[28] Higgins JPT, Thompson SG, Deeks JJ, Altman DG (2003). Measuring inconsistency in meta-analyses. BMJ.

